# Research on Evaluating the Filtering Method for Broiler Sound Signal from Multiple Perspectives

**DOI:** 10.3390/ani11082238

**Published:** 2021-07-29

**Authors:** Zhigang Sun, Mengmeng Gao, Guotao Wang, Bingze Lv, Cailing He, Yuru Teng

**Affiliations:** 1 Electronic Engineering College, Heilongjiang University, Harbin 150080, China; 2191313@s.hlju.edu.cn (Z.S.); 2191376@s.hlju.edu.cn (M.G.); 2201652@s.hlju.edu.cn (B.L.); 2201742@s.hlju.edu.cn (C.H.); 2201746@s.hlju.edu.cn (Y.T.); 2 School of Electrical Engineering and Automation, Harbin Institute of Technology, Harbin 150001, China

**Keywords:** multiple perspectives, signal-to-noise ratio, root mean square error, prediction accuracy, signal filtering method, broiler sound signal

## Abstract

**Simple Summary:**

Broiler sound signals can reflect their own health status, such as crowing when they are hungry, coughing when they are in pain or ill, purring when there are foreign bodies in their throat, and flapping wings when they are fighting with each other. Many studies have been carried out to improve animal welfare based on this. However, the reality is that in addition to the emotional sounds mentioned above, there still exists back noise in the broiler sound signal collected in the farm, such as the noise of people walking and ventilation equipment. Therefore, it is necessary to adopt effective signal filtering methods to filter out these noises, and further obtain high-quality broiler sound signals for animal welfare research. In this study, five of the most classic and effective signal filtering methods were used to filter the back noise in broiler sound signals, and different evaluation indicators from two angles were used for “scoring” the filtering effect of each method in order to select the outstanding performers. These studies have laid the foundation for the follow-up study of broiler health monitoring.

**Abstract:**

Broiler sounds can provide feedback on their own body condition, to a certain extent. Aiming at the noise in the sound signals collected in broiler farms, research on evaluating the filtering methods for broiler sound signals from multiple perspectives is proposed, and the best performer can be obtained for broiler sound signal filtering. Multiple perspectives include the signal angle and the recognition angle, which are embodied in three indicators: signal-to-noise ratio (SNR), root mean square error (RMSE), and prediction accuracy. The signal filtering methods used in this study include Basic Spectral Subtraction, Improved Spectral Subtraction based on multi-taper spectrum estimation, Wiener filtering and Sparse Decomposition using both thirty atoms and fifty atoms. In analysis of the signal angle, Improved Spectral Subtraction based on multi-taper spectrum estimation achieved the highest average SNR of 5.5145 and achieved the smallest average RMSE of 0.0508. In analysis of the recognition angle, the kNN classifier and Random Forest classifier achieved the highest average prediction accuracy on the data set established from the sound signals filtered by Wiener filtering, which were 88.83% and 88.69%, respectively. These are significantly higher than those obtained by classifiers on data sets established from sound signals filtered by other methods. Further research shows that after removing the starting noise in the sound signal, Wiener filtering achieved the highest average SNR of 5.6108 and a new RMSE of 0.0551. Finally, in comprehensive analysis of both the signal angle and the recognition angle, this research determined that Wiener filtering is the best broiler sound signal filtering method. This research lays the foundation for follow-up research on extracting classification features from high-quality broiler sound signals to realize broiler health monitoring. At the same time, the research results can be popularized and applied to studies on the detection and processing of livestock and poultry sound signals, which has extremely important reference and practical value.

## 1. Introduction

Relevant studies have shown that animal sounds contain a lot of emotional information, such as crowing when they are hungry [1], coughing when they are in pain or ill [2,3], purring when there are foreign bodies in their throat, and flapping wings when they are fighting with each other [4]. Therefore, sounds can be used to obtain feedback on the animal’s own body conditions and emotion changes and can be used as an auxiliary method to evaluate animal welfare [5]. Compared with traditional physiological index detection, such as obtaining animal blood for detection, the sound detection method has the advantages of no stress, no contact, and continuous collection. Therefore, researchers can evaluate the comfort level of animals’ living environment and health status by collecting their sounds in the farm and carry out further measures to improve animal welfare [6,7]. Many researchers have carried out related research on this. For example, Marx G et al. [8] studied chicks’ voices in their grouping process, including distress calls, short peeps, warblers, and pleasure notes, and found that the chicks’ voices would change with their growth environment and social skills. Aydin, A. et al. [9] studied broiler feed intake and designed an advanced sound detection system for detecting broilers’ continuous pecking behavior. The system used real-time sound processing technology to accurately measure the broilers’ feed intake at the group level. Carpentier, Lenn et al. [10] studied chicken sneezes under different noise sources. By extracting the features that can characterize a chicken’s sneezes, the number of sneezes in a fixed-duration chicken sound signal can be monitored, and then the chicken’s health can be obtained. Yang et al. [11] studied sounds from different sources in commercial broiler houses. By using the Maximum Overlap Discrete Wavelet Transform (MODWT), the Fast Fourier Transform (FFT) method, and the Gaussian regression model, the sound frequency ranges of six common sounds, including bird vocalization, fan, feed system, heater, flapping wing, and dustbathing, were determined. Du et al. [12] developed a monitoring system by using a Kinects microphone array. It is a feasible method to detect the abnormal situation of poultry at night by monitoring the number and regional distribution of sounds. Huang et al. [13] proposed a new detection method for bird flu based on audio analysis. By analyzing and processing the frequency domain of the extracted broiler sound signal, the Mel-Frequency Cepstral Coefficients (MFCC) was determined to be used as the distinguishing standard of healthy and infected broilers, and a parameter-optimized Support Vector Machine (SVM) was used to quickly predict broiler health.

It can be seen that broiler sound signals are the basis of various studies. Therefore, obtaining high-quality broiler sound signals is very important to carry out research on broilers’ growth condition and health status. Many researchers have used various signal filtering methods to process the sound signals collected in the farms in order to obtain a reliable data source for following research. For example, in his research mentioned above, Carpentier, Lenn et al. [10] used Spectral Subtraction to filter the sound signal before extracting features from broilers’ sneezes. Cheng et al. [14] proposed a new broiler sound recognition method based on Sparse Representation. Specifically, it used Sparse Representation to reconstruct the sound signal, to complete signal filtering and feature extraction, and also used the SVM as the classification model to recognize the broiler sound in different environments. Aiming at the noise generated by fans and other ventilation equipment in the perch breeding mode, Yu et al. [15] used a variety of signal filtering methods to remove fan noise from the laying hens’ sound signal and used a pattern recognition algorithm to identify the specific sound type from the filtered sound signal.

The research of broiler sound signal filtering method is still being explored. In recent years, the author’s research group has been committed to introducing machine learning methods into the research of signal classification and recognition [16,17,18,19,20,21], and has further applied the research results to conduct research on broiler health monitoring, which realized effective recognition of a fixed-duration sound signal collected in the broiler farm by constructing a classifier with good performance; i.e., one that is able to identify the specific sound types and their numbers in a fixed-duration sound signal, including crows, coughs, purrs, and flapping wings. By calculating the ratio of the number of coughs to the number of all sound types, the cough rate can be obtained. Then, we can judge the broilers’ health in the current area by comparing the value of the cough rate. It can be seen that the prediction performance of the classifier directly determines the judgment of the broilers’ health. The data set for training the classifier is extracted from a large number of broiler sound signals. Therefore, the quality of the sound signal determines the quality of the data set, and the quality of the data set affects the prediction performance of the classifier. It can be seen that the broiler sound signal filtering method proposed in this study is an important element which is used to efficiently filter the collected sound signals to form a high-quality data set. In addition, this research combined the signal-to-noise ratio (SNR) and the root mean square error (RMSE) to comprehensively evaluate the processing effects of different methods from the signal angle, and prediction accuracy was used to evaluate their processing effects from the recognition angle. Finally, both the signal angle and the recognition angle were considered comprehensively, and the best broiler sound signal filtering method was obtained.

## 2. Methods

On the basis of summarizing the previous studies and consulting a large amount of literatures, this article took five signal filtering methods as the research objects, including Basic Spectral Subtraction, Improved Spectral Subtraction based on multi-taper spectrum estimation, Wiener filtering, and Sparse Decomposition using thirty atoms and fifty atoms [22,23,24,25,26], to evaluate their filtering effect on broiler sound signal from multiple perspectives. In this chapter, the author briefly describes the principles of the five methods.

### 2.1. Basic Spectral Subtraction

Basic Spectral Subtraction is a signal filtering method introduced in the early days. It is based on the assumption of additive noise and obtains a pure signal by subtracting noise components from a noisy signal [27]. Suppose that the time sequence of the sound signal is x(n), the i-th frame signal after windowing and framing is $x_{i}\left(m\right)$, and the frame length is *N*. Any frame signal $x_{i}\left(m\right)$ after Fast Fourier Transform (FFT) is:

(1) $$X_{i}\left(k\right)=\sum_{m=0}^{N-1}x_{i}\left(m\right)exp\left(-j\frac{2\pi mk}{N}\right)\;\;\;\;\;k=0,1,\ldots ,N-1$$

Calculate the absolute value of $X_{i}\left(k\right)$, obtain the amplitude of each frame signal as $\left|X_{i}\left(k\right)\;\right|$, and then calculate its phase angle as:

(2) $$X_{angle}^{i}\left(k\right)=arctan\left[\frac{lm\left(X_{i}\left(k\right)\right)}{Re\left(X_{i}\left(k\right)\right)}\right]$$

Save the amplitude and phase angle of each frame signal. Knowing that the duration of the preamble silent frame (noise segment) is *IS*, and the corresponding frame number is *NIS*, the average energy value of the noise segment can be calculated as:

(3) $$D\left(k\right)=\frac{1}{NIS}\sum_{i=1}^{NIS}|X_{i}\left(k\right){|}^{2}$$

The calculation formula of Basic Spectral Subtraction is further obtained as:

(4) $$|{\hat{X}}_{i}\left(k\right){|}^{2}=\{\begin{array}{c} \;\left|X_{i}\left(k\right){|}^{2}-a\times D\left(k\right)\;\;\;\;\;\right|X_{i}\left(k\right){|}^{2}\;\geq \;a\times D\left(k\right)\\ b\times \left|X_{i}\left(k\right){|}^{2}\;\;\;\;\;\;\;\;\;\;\;\;\;\;\;\;\;\;\;\;\;\;\right|X_{i}\left(k\right){|}^{2}<a\times D\left(k\right) \end{array}$$

In the formula, *a* and *b* are two constants; *a* is an over-subtraction factor, and *b* is a gain compensation factor.

**Figure 1:**
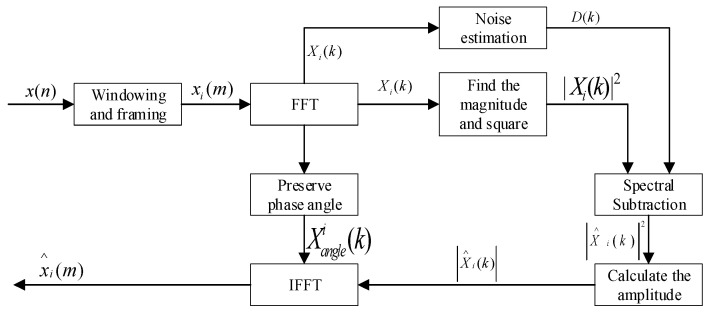
Schematic diagram of Basic Spectral Subtraction.

After the Spectral Subtraction is obtained, the amplitude of each frame signal is $\left|{\hat{X}}_{i}\left(k\right)\right|$, and the phase angle of each frame signal is $X_{angle}^{i}\left(k\right)$. By performing Inverse Fast Fourier Transform (IFFT) on $\left|{\hat{X}}_{i}\left(k\right)\right|$ and $X_{angle}^{i}\left(k\right)$, the signal sequence after Basic Spectral Subtraction filtering can be obtained as ${\hat{x}}_{i}\left(m\right)$. Among them, the insensitivity of sound signal to the phase is used, and the phase angle information before spectral subtraction is directly used in the signal after spectral subtraction. The principle of Basic Spectral Subtraction is shown in Figure 1.

### 2.2. Improved Spectral Subtraction

In Basic Spectral Subtraction, the periodogram method is commonly used when estimating the power spectrum of the signal, while the traditional periodogram method uses only one data window [28]. However, in the process of using this method, it will inevitably bring about a large estimation deviation and estimation variance, resulting in the enhanced signal containing large amounts of noise with certain rhythmic fluctuations and sounds similar to music [29]. For this reason, Thomson [30] proposed an improved multi-taper spectrum estimation method in 1982, it used multiple orthogonal data windows to obtain a direct spectrum for the same data sequence, and then averaged them to obtain the spectrum estimate, and thus a smaller estimate variance could be obtained. Therefore, the multi-taper spectrum is a more accurate spectrum estimation method than the periodogram method, and using Improved Spectral Subtraction method based on it can effectively reduce the “music noise” in the signal.

The definition of the multi-taper spectrum is:

(5) $$S^{mt}\left(\omega \right)=\frac{1}{L}\sum_{k=0}^{L-1}S_{k}^{mt}\left(\omega \right)$$

In the formula, *L* is the number of data windows and $S^{mt}$ is the spectrum of the k-th data window, which is defined as:

(6) $$S_{k}^{mt}\left(\omega \right)=|\sum_{n=0}^{N-1}a_{k}\left(n\right)x\left(n\right)e^{-jn\omega }{|}^{2}$$

In the formula, x(n) is the signal sequence and *N* is the sequence length. $a_{k}\left(n\right)$ is the k-th data window function, which meets the mutual orthogonality between data windows.

(7) $$\begin{array}{c} \sum a_{k}\left(n\right)a_{j}\left(n\right)=0\;\;\;\;\;\\ \sum a_{k}\left(n\right)a_{j}\left(n\right)=1\;\;\;\;\; \end{array}\begin{array}{c} k\neq j\\ k=j \end{array}\}$$

Among them, the data window is a set of orthogonal discrete ellipsoid sequences, also called Slepian windows. The specific steps of Improved Spectral Subtraction based on multi-taper spectrum estimation are as follows.

Step 1: As known that the time sequence of the sound signal is x(n), the i-th frame signal after windowing and framing is $x_{i}\left(m\right)$, and there is overlap between adjacent frames.

Step 2: Perform FFT on the frame signal $x_{i}\left(m\right)$, calculate the amplitude spectrum $\left|X_{i}\left(k\right)\right|$ and phase spectrum $\theta_{i}\left(k\right)$ respectively, and perform smoothing between adjacent frames to calculate the average amplitude spectrum $\left|{\bar{X}}_{i}\left(k\right)\right|$ as:

(8) $$\left|{\bar{X}}_{i}\left(k\right)\right|=\frac{1}{2M+1}\sum_{j=-M}^{M}\left|X_{i+j}\left(k\right)\right|$$

Take the i-th frame signal as the center, and take *M* frame signals before and after, so there are 2M+1 frame signals for mean value calculation. In practice, *M* is usually taken as one, that is, the average is carried out in three frame signals.

Step 3: Perform multi-taper spectrum estimation on the frame signal $x_{i}\left(m\right)$ to obtain the multi-taper spectrum power spectrum density P(k,i) (i represents the i-th frame signal, k represents the k-th spectral line):

(9) $$P\left(k,i\right)=PMTM\left[x_{i}\left(m\right)\right]$$

In the formula, *PMTM* is the multi-taper spectrum power spectral density estimation of the signal.

Step 4: The multi-taper spectrum power spectrum density estimation value is also smoothed between adjacent frames, and the smooth power spectrum density $P_{y}\left(k,i\right)$ is calculated as:

(10) $$P_{y}\left(k,i\right)=\frac{1}{2M+1}\sum_{-M}^{M}P\left(k,i+j\right)$$

Similarly, take the i-th frame signal as the center, and take *M* frame signals before and after, so there are 2M+1 frame signals for mean calculation. In practice, *M* is usually taken as one, that is, the average is carried out in three frame signals.

Step 5: Knowing that the duration of the preamble silent frame (noise segment) is *IS*, and the corresponding frame number is *NIS*, the average power spectral density value of the noise can be calculated as $P_{n}\left(k\right)$. The calculation formula is:

(11) $$P_{n}\left(k\right)=\frac{1}{NIS}\sum_{i=1}^{NIS}P_{y}\left(k,i\right)$$

Step 6: The gain factor g(k,i) is calculated by using the spectral subtraction relationship as:

(12) $$g\left(k,i\right)=\{\begin{array}{c} \left(P_{y}\left(k,i\right)-\alpha P_{n}\left(k\right)\right)/P_{y}\left(k,i\right)\;\\ \beta P_{n}\left(k\right)/P_{y}\left(k,i\right) \end{array}\;\begin{array}{c} \;\;\;\;\;P_{y}\left(k,i\right)-\alpha P_{n}\left(k\right)\geq 0\\ \;\;\;\;\;P_{y}\left(k,i\right)-\alpha P_{n}\left(k\right)<0 \end{array}$$

In the formula, α and β are two constants, α is an over-subtraction factor, and β is a gain compensation factor. Properly selected, α can effectively remove music noise, but too large a value of α will cause sound distortion.

Step 7: Through the gain factor g(k,i) and the average amplitude spectrum $\left|{\bar{X}}_{i}\left(k\right)\right|$, the amplitude spectrum after spectrum subtraction can be obtained as:

(13) $$\left|{\hat{X}}_{i}\left(k\right)\right|=g\left(k,i\right)\times \left|{\bar{X}}_{i}\left(k\right)\right|$$

Combine the subtracted amplitude spectrum $\left|{\hat{X}}_{i}\left(k\right)\right|$ with the phase spectrum $\theta_{i}\left(k\right)$ in step 2 to perform IFFT. Restore the frequency domain $\left|{\hat{X}}_{i}\left(k\right)\right|$ to the time domain to obtain the noise-reduced sound signal ${\hat{x}}_{i}\left(m\right)$ as:

(14) $${\hat{x}}_{i}\left(m\right)=IDFT\left[\left|{\hat{X}}_{i}\left(k\right)\right|exp\left[j\theta_{i}\left(k\right)\right]\right]$$

**Figure 2:**
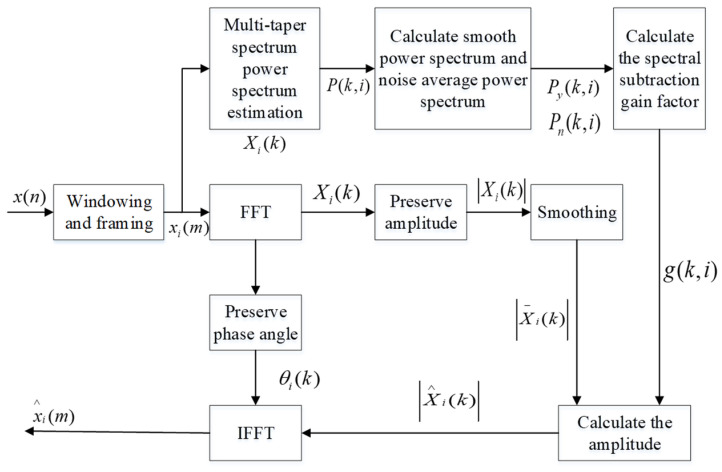
Schematic diagram of Improved Spectral Subtraction.

The principle of Improved Spectral Subtraction is shown in Figure 2.

### 2.3. Wiener Filtering

Wiener filtering is based on the statistical independence of sound and noise, and uses the Minimum Mean Square Error (MMSE) criterion for signal filtering [31,32]. Suppose the noisy sound signal is:

(15) y(n)=s(n)+d(n)

In the formula, s(n) and d(n) represent pure sound signal and noise, respectively. Wiener filtering is to design a filter h(n) for which, when the input is y(n), the output is:

(16) $$\hat{s}\left(n\right)=y\left(n\right)\times h\left(n\right)=\sum_{m=-\infty }^{+\infty }y\left(n-m\right)h\left(m\right)$$

In the formula, $\hat{s}\left(n\right)$ minimizes the mean square error $\varepsilon =E\left[{\left\{s\left(n\right)-\hat{s}\left(n\right)\right\}}^{2}\right]$ of s(n) and d(n) according to MMSE.

According to the principle of orthogonality, the best h(n) must satisfy Equation (17) for all m:

(17) $$E\left[\left\{s\left(n\right)-\hat{s}\left(n\right)\right\}\cdot y\left(n-m\right)\right]=0$$

Incorporating Equation (16) into Equation (17) and taking the Fourier Transform, the spectrum estimator of Wiener filtering can be derived as:

(18) $$H\left(k\right)=\frac{P_{s}\left(k\right)}{P_{s}\left(k\right)+P_{d}\left(k\right)}$$

**Figure 3:**
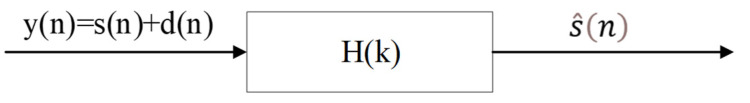
The input–output relationship of Wiener filtering. y(n): the input signal, s(n): the pure signal, d(n): noises, H(k): the wiener filtering spectrum estimator, $\hat{s}\left(n\right)$: the output signal.

In the formula, $P_{s}\left(k\right)$ is the power spectral density of sound signal, and $P_{d}\left(k\right)$ is the power spectral density of noise. The signal filtering is realized by minimizing the mean square error of $|x\left(n\right)-\hat{x}\left(n\right){|}^{2}$. The above-mentioned Wiener filtering process (input-output relationship) is shown in Figure 3.

Based on this, the specific implementation steps of Wiener filtering are as follows:

Step 1: As known that the time sequence of the sound signal is x(n), the i-th frame signal after windowing and framing is $x_{i}\left(m\right)$, and there is overlap between adjacent frames as well.

Step 2: Perform FFT on the frame signal $x_{i}\left(m\right)$, calculate the amplitude spectrum $\left|X_{i}\left(k\right)\right|$ and phase spectrum $\theta_{i}\left(k\right)$, respectively, save them, and further calculate the power spectrum as:

(19) $$P_{y}\left(k,i\right)=|X_{i}\left(k\right){|}^{2}$$

Step 3: Knowing that the duration of the preamble silent frame (noise segment) is IS, and the corresponding frame number is NIS, the average power spectral density of the noise segment can be calculated as $P_{n}\left(k\right)$, and the average noise amplitude spectrum can be calculated as $\left|{\bar{X}}_{n}\left(k,i\right)\right|$:

(20) $$P_{n}\left(k\right)=\frac{1}{NIS}\sum_{i=1}^{NIS}P_{y}\left(k,i\right)=\lambda_{d}\left(k\right)$$

(21) $${\bar{X}}_{n}\left(k,i\right)=\frac{1}{NIS}\sum_{i=1}^{NIS}\left|X_{i}\left(k\right)\right|$$

Step 4: We use endpoint detection to obtain segment and non-segment frames. For segment frames, we modify the average noise power spectrum (variance) and the average noise amplitude spectrum. Among them, the average power spectrum of noise is $\lambda_{d}\left(k\right)$. For non-segment frames, the prior SNR ${\hat{\xi }}_{i}\left(k\right)$ and the posterior SNR $\gamma_{i}\left(k\right)$ are calculated first, and then the transfer function $H_{i}\left(k\right)$ of Wiener filtering is determined.

(22) $$\gamma_{i}\left(k\right)=\frac{{\left|Y_{i}\left(k\right)\right|}^{2}}{\lambda_{d}\left(k\right)}$$

In the formula, $Y_{i}\left(k\right)$ is the spectral value of the noisy signal at the corresponding frequency point.

(23) $${\hat{\xi }}_{i}\left(k\right)=\alpha \xi_{i-1}\left(k\right)+\left(1-\alpha \right)\left(\gamma_{i}\left(k\right)-1\right)$$

In the formula, $\xi_{i}\left(k\right)=\frac{E\left[{\left|S_{i}\left(k\right)\right|}^{2}\right]}{\lambda_{d}\left(k\right)}$, $S_{i}\left(k\right)=H_{i}\left(k\right)\cdot Y_{i}\left(k\right)$, $H_{i}\left(k\right)=\frac{{\hat{\xi }}_{i}\left(k\right)}{{\hat{\xi }}_{i}\left(k\right)+1}$.

Step 5: Calculate the output ${\hat{S}}_{i}\left(k\right)$ of each frame signal after Wiener filtering as:

(24) $${\hat{S}}_{i}\left(k\right)=H_{i}\left(k\right)\cdot Y_{i}\left(k\right)$$

Step 6: After performing Inverse Fourier Transform (IFT) on the amplitude spectrum ${\hat{S}}_{i}\left(k\right)$ and the phase spectrum $\theta_{i}\left(k\right)$, the sound signal restored to the time domain can be obtained, which is the filtered signal ${\hat{x}}_{i}\left(m\right)$:

(25) $${\hat{x}}_{i}\left(m\right)=IDFT\left[{\hat{S}}_{i}\left(k\right)exp\left[j\theta_{i}\left(k\right)\right]\right]$$

### 2.4. Sparse Decomposition

Analyzing from the perspective of sparse decomposition, the noisy signal contains two parts, namely the signal and the noise, and the signal is the sparse component in the noisy signal. The signal has a certain structure, and its structural characteristics are consistent with the atomic characteristics. While noise is random and uncorrelated, so it has no structural characteristics [33]. If the meaningful (the enough larger energy) atoms can be extracted from the signal, the extracted part will be used as the signal. If we cannot continue to extract meaningful atoms from the signal residual, it is considered that all the signal residuals are noises.

In the iterative process of signal sparse decomposition, each decomposition iterative process selects the atomic vector $g_{r_{k}}$ with the largest inner product of the signal or signal residual $R^{k}f$. This is actually equivalent to the maximum correlation between the atomic vector $g_{r_{k}}$ and the signal or residual signal $R^{k}f$, and $g_{r_{k}}$ is the closest to $R^{k}f$. Sparse Decomposition is the process of continuously tracking and extracting the atomic vector that best matches the original signal and its residual signal. These extracted atomic vectors can be regarded as the components of the original signal.

The key to performing signal filtering by using Sparse Decomposition is how to determine what kind of atom represents the signal, which is meaningful. In the signal sparse decomposition process based on the Matching Pursuit (MP) algorithm, this problem is the determination of the iterative stop condition. Generally speaking, there are two ways to determine the stop of iteration. One method is to use hard thresholds. That is, the iterative stop condition of Sparse Decomposition is set as the upper limit M of the number of iterations, and then the linear combination of M atoms is used to approximate the original noisy signal, and the approximate representation of the signal is taken as the denoised signal of the original noisy signal, and the residual signal is used as noise. Another method is the coherence ratio threshold method. That is, the sparse decomposition iteration stop condition is set as a lower limit of the coherence ratio [34].

**Figure 4:**
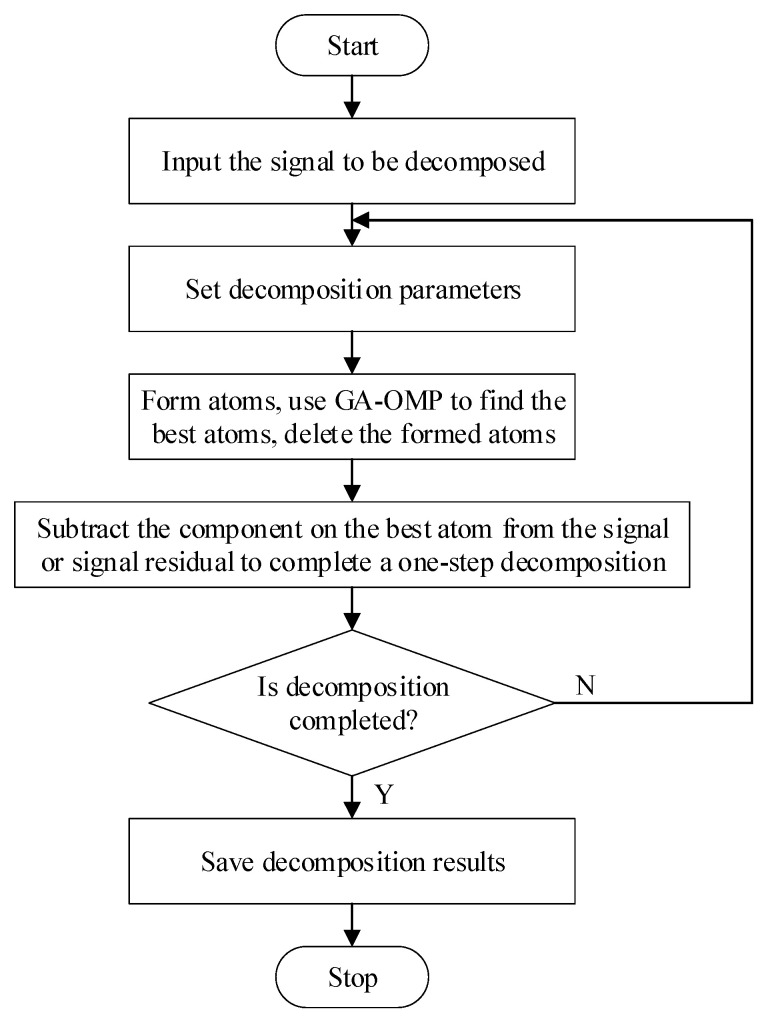
Process of sparse decomposition by using GA-OMP.

The Orthogonal Matching Pursuit (OMP) algorithm based on a Genetic Algorithm (GA), which is continuously developed on the basis of the MP algorithm, was determined as Sparse Decomposition method specifically used in this article, and the first iterative stopping method was also determined. Through the summary of a large number of previous experiments, Sparse Decomposition method of using thirty atoms and fifty atoms was determined to achieve better filtering effects than using other atom numbers. The process of Sparse Decomposition by using GA-OMP is shown in Figure 4.

When GA-OMP performs signal reconstruction, in order to better describe the time-varying characteristics of the signal, a complete time frequency atomic dictionary is usually used. Since the Gabor dictionary has better time frequency characteristics, we chose it as the over-complete dictionary in this article. The formula is:

(26) $$g_{r}\left(t\right)=\frac{1}{\sqrt{s}}g\left(\frac{t-\mu }{s}\right)cos\left(vt+\omega \right)$$

In the formula, $g_{r}\left(t\right)=e^{-\pi t^{2}}$ is the Gaussian window function, γ=(s,μ,v,ω) is the time frequency parameter, s is the expansion factor, μ is the translation factor, v is the frequency factor, and ω is the phase factor.

The space of time frequency parameters can be separated into $\gamma =\left(\alpha^{j},p\alpha^{j}∆\mu ,k\alpha^{-j}∆v,i∆\omega \right)$. In the formula, α=2, ∆μ=1/2, ∆v=π, ∆ω=π/6, $0<j\;\leq \;log_{2}^{N}$, $0\;\leq \;p<N2^{-j+1},\;0\;\leq \;k<2^{j+1}$, 0≤i≤12, ***N*** is the number of samples in each frame.

## 3. Verification and Analysis

The broiler sound signals were collected in the No. 3 breeding shed in the Huixing Farm (44°91′ N, 130°02′ E), Liushu Town, Linkou County, Mudanjiang City, Heilongjiang Province on 18 December 2020. The basic characteristics of the No. 3 breeding shed are: east-west direction, 122.0 m long, 18.8 m wide, roof height is 4.6 m, single-storey, and the layout of the shed is four rows of walkways. At the westernmost entrance of the shed, there is a reserved space with the length of 3.2 m and the width of 18.8 m, which is used for walking and placing breeding feed. The author used a fence to enclose an area of 1.2 m long and 2 m wide in the southwest corner as the experimental area and covered it with 50 to 100 mm thick wood chips as bedding. This area is about two meters away from the broiler colony breeding area. It should be noted that because the sound pitch of broilers is small, and multiple broilers will vocalize at the same time, it is difficult to directly use the audio collection system to collect the broiler sound signals in the broiler group breeding area. Most of these sound signals overlap, which is not conducive to the development of this research. Therefore, the author built an experimental area far away from the broiler group breeding area for the successful collection of broiler sound signals. In this way, the problem of overlap broiler sound signals had been solved, and the built experimental area under the same noisy environment. The noise here includes the sound made by breeders walking in the walkway, and by the vertically placed ventilation equipment.

The specific breed of broiler in the farm is white-feather broiler. Generally, their incubation period is twenty-one days (about three weeks) and the growth period is fifty days (about seven weeks). The test subjects selected by the author are four white-feather broilers of the same batch of eight weeks old, of which two are healthy and the other two are diseased. The author put them into the experimental area one by one and used the audio collection system to collect sound signals. The audio collection system used the chassis of National Instruments Co., Ltd. (Austin, TX, U.S.), and its specific model is PXI-1050. For the chassis, the model of the internal controller is PXI-8196, the model of the sound capture card is NI4472B (8-channels synchronous acquisition, 24 bits resolution, 102.4 kS/s sampling frequency). The sound sensor selects the model of MPA201 from BSWA TECHNOLOGY CO., LTD (Beijing, China), the response frequency is 20 Hz to 20,000 Hz, and the sensitivity is 50 mV/Pa. The sound recording software chooses NI Sound and Vibration Assistant 2010, the sampling frequency is 32 kHz and the sampling accuracy is 16 bits, and monophonic acquisition can be performed. The audio collection system collects fixed-duration sound signals in a time unit of five minutes, the interval between two adjacent sound signals is thirty seconds, and the collected sound signals are stored in the computer in the “*.tdms” format. From reference [35], the specific sound collection and procession information mentioned above can be seen. For each five-minute sound signal, the author performed pulse extraction and endpoint detection in MATLAB. By setting the detection threshold to be 1.5 times the total energy of the leading non-segment frame, the effective signal pulse can be extracted. Through the artificial recognition of the broiler sound signals saved after pulse extraction and endpoint detection, the specific sound types that can be detected include crows, coughs, purrs, and flapping wings. Among them, the crow is the natural short cheeping during the growth of healthy broilers, and this sound is relatively loud and sharp. The cough is the prolonged abnormal croak of diseased broilers, and this sound is relatively low. The purr is the snoring sound made when the broiler is asleep, and this sound is continuously fluctuating. The flapping wings sound is produced by inciting the friction and vibration between the wings and the air when the broiler is moving. The sound amplitude is large and the continuous time is long.

The author chose ten sound signals with better recording effects (that is, the sound signal contains as many crows, coughs, purrs, and flapping wings as possible), and used the audio processing software Adobe Audition 14.0 to intercept the ten-second-duration sound signals from each of the selected ten sound signals, which were used as the data source for the verification and analysis of this research. On this basis, the author designed the software programs of Basic Spectral Subtraction, Improved Spectral Subtraction based on multi-taper spectrum estimation, Wiener filtering, Sparse Decomposition using thirty atoms and fifty atoms, running on MATLAB, and processed the intercepted ten sound signals separately. The sound signals processed by the five signal filtering methods were evaluated from both the signal angle and the recognition angle, and the best-performing method was obtained through comprehensive analysis.

### 3.1. Multiple Perspective Evaluation Indicators

From the signal angle, this article mainly selects two indicators, signal-to-noise ratio (SNR) and root mean square error (RMSE), to evaluate the processing effects of five filtering methods. Suppose the noisy audio signal is x(n)=s(n)+d(n), where x(n) is the sound signal captured by voice recorder, and s(n) and d(n) represent the pure signal and noise, respectively. Then, the SNR refers to the ratio of signal to noise in a fixed-duration sound signal. Often, the larger the SNR of the sound signal processed by a certain signal filtering method, the better the processing effect of the current method. Its expression is:

(27) $$SNR=10lg\frac{\sum_{n=0}^{N}s{\left(n\right)}^{2}}{\sum_{n=0}^{N}{\left[x\left(n\right)-s\left(n\right)\right]}^{2}}$$

In the formula, $\sum_{n=0}^{N}s{\left(n\right)}^{2}$ represents the energy of the sound signal, $\sum_{n=0}^{N}{\left[x\left(n\right)-s\left(n\right)\right]}^{2}$ represents the energy of the noise.

The RMSE refers to the root mean square of the variance between the sound signal before and after signal filtering. Often, the smaller the RMSE of the sound signal processed by a certain signal filtering method, the closer the filtered sound signal is to the original noise-free signal and the better the processing effect of the current method. Its expression is:

(28) $$RMSE=\sqrt{\frac{1}{N}\sum_{n=0}^{N}{\left[x\left(n\right)-f\left(n\right)\right]}^{2}}$$

In the formula, x(n) is the sound signal before filtering, and f(n) is the sound signal after filtering.

From the recognition angle, this article extracted 39-dimensional MFCC features from the sound signals processed by five signal filtering methods, thereby we established five data sets for training classifiers accordingly. In this article, the kNN classifier and the Random Forest classifier under the default parameter configuration were used to make predictions on the five data sets separately, and five prediction accuracies were obtained. Therefore, on which data set the classifier achieved the highest prediction accuracy indicated that the overall quality of that data set was the best, further indicating that the quality of the sound signal that constitutes the data set is the best, and the processing effect of the corresponding signal filtering method is the best.

Suppose the data set is $D=\left\{\left(x_{1},y_{1}\right),\left(x_{2},y_{2}\right),\cdots ,\left(x_{m},y_{m}\right)\right\}$, where $y_{i}$ is the true label corresponding to the data $x_{i}$, and $f\left(x_{i}\right)$ is the label predicted by the classifier f. The prediction accuracy can be expressed as the ratio of the number of data that are correctly predicted to the total number of data, that is:

(29) $$acc\left(f:D\right)=\frac{1}{m}\sum_{i=1}^{m}I\left(f\left(x_{i}\right)=y_{i}\right)$$

In the formula, I is the indicator function, and when $f\left(x_{i}\right)=y_{i}$, $I\left(f\left(x_{i}\right)=y_{i}\right)=1$.

### 3.2. Signal Angle

The author designed the software programs of Basic Spectral Subtraction, Improved Spectral Subtraction based on multi-taper spectrum estimation, Wiener filtering, and Sparse Decomposition running in MATLAB. Among them, the software program of Sparse Decomposition method contains using thirty atoms and fifty atoms. It should be noted that the selection of thirty atoms and fifty atoms is based on the author’s extensive research in the previous period. In this article, five signal filtering methods were performed on the same intercepted ten sound signals, and the SNR and RMSE of the processed sound signals were calculated. The calculation results are shown in Table 1.

In Table 1, the BSS represents Basic Spectral Subtraction, the ISS represents Improved Spectral Subtraction based on multi-taper spectrum estimation, the WF represents Wiener filtering, and the SD represents Sparse Decomposition using different atoms. The above abbreviations will not be repeated in the following table.

It can be seen from Table 1 that the SNR obtained on the sound signal that was filtered by using Improved Spectral Subtraction is greater than that obtained by using Basic Spectral Subtraction. The RMSE obtained on the sound signal that was filtered by using Improved Spectral Subtraction is slightly smaller than that obtained by using Basic Spectral Subtraction. Considering that the larger the SNR or the smaller the RMSE of the sound signal before and after signal filtering, the better the filtering effect, from the signal angle, the filtering effect of Improved Spectral Subtraction is therefore better than that of Basic Spectral Subtraction, which is in line with theoretical reasoning. At the same time, the SNR obtained on the sound signal that was filtered by using fifty-atom Sparse Decomposition is greater than that obtained by using thirty-atom Sparse Decomposition. The RMSE obtained on the sound signal that was filtered by using fifty-atom Sparse Decomposition is smaller than that obtained by using thirty-atom Sparse Decomposition. It is also concluded that fifty-atom Sparse Decomposition is better than thirty-atom Sparse Decomposition. Considering from the principle perspective, this shows that, while using thirty atoms, it not only sparse the noise in the noisy signal, but also sparse a part of the original noise-free signal. The SNR obtained on the sound signal that was filtered by using Wiener filtering is between the two Spectral Subtraction methods and the two Sparse Decomposition methods. From comprehensive analysis of the signal angle, Improved Spectral Subtraction based on multi-taper spectrum estimation has achieved the largest SNR and the smallest RMSE, indicating that this method has achieved the best filtering effect.

### 3.3. Recognition Angle

As mentioned above, five signal filtering methods were performed on the same intercepted ten sound signals, but we no longer calculate their SNR and RMSE at this time. Instead, for each processed sound signal, the author first performed framing processing on it, and then combined the endpoint detection method to obtain the start frame and end frame of one single specific sound type in one sound signal, and the combination of the start frame and the end frame of multiple specific sound types in one sound signal can be finally obtained. For example, after the sound signal framing processing and endpoint detection is completed, the 10-th frame is the start frame of the crow, and the 55-th frame is the end frame. This is the first specific sound type in this sound signal. It can also be obtained that the 67-th frame is the start frame of the cough, and the 135-th frame is the end frame. This is the second specific sound type in this sound signal. By analogy, multiple specific sound types in this sound signal can be obtained. For each specific sound type, pieces of feature data can be extracted from all signal frames between its start frame and end frame. Therefore, a total of forty-six feature data can be extracted from the 10-th frame to the 55-th frame, and the label of the data is “crow”, and a total of sixty-eight feature data can be extracted from 67-th frame to 135-th frame, and also the label of the data is “cough”. By analogy, feature data corresponding to multiple specific sound types in this sound signal can be obtained. By merging the feature data of the same label, multiple feature data corresponding to the four labels (crow, cough, purr, and flapping wing) can be extracted from one intercepted sound signal. Similarly, for ten intercepted sound signals processed by the same filtering method, multiple feature data corresponding to four labels can also be extracted from the ten processed sound signals to form the data set corresponding to the current filtering method. Therefore, this research can finally form five data sets corresponding to five signal filtering methods, and their specific description is shown in Table 2. It should be noted that the label of the data is marked by manual identification. Specifically, we invited experienced breeders to identify and record the specific sound types that appear in chronological order in the ten sound signals to ensure the reliability of data labeling.

**Figure 5:**
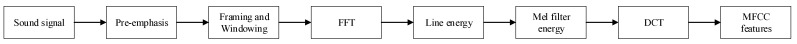
Block diagram of MFCC feature extraction.

It should be noted that the features extracted from each frame signal are the 39-dimensional MFCC features. The MFCC used here is a cepstrum parameter extracted from the Mel-Frequency domain. It first maps the linear spectrum of the signal to the Mel nonlinear spectrum based on auditory perception, and then converts it to the cepstrum [36,37]. Equation (29) is to convert from frequency to Mel scale, and Equation (30) is to return from Mel scale to frequency. The block diagram of MFCC feature extraction is shown in Figure 5.

(30) $$f_{mel}=2595log_{10}\left(1+f/700\right)$$

(31) $$f=700\left(10^{\frac{f_{mel}}{2595}}-1\right)$$

As shown in Figure 5, we generally take the 1-st to 13-th cepstrum coefficients after Discrete Cosine Transform (DTC) as the standard 13-dimensional MFCC parameters, which reflect the static characteristics of the sound signal. The dynamic characteristics of the sound signal can be obtained by the difference of the static characteristics. The first-order difference of static characteristics reflects the speed of the sound signal change, while the second-order difference reflects the acceleration of the sound signal change. We combined the standard MFCC parameters with the first-order difference and the second-order difference to obtain a total of 39-dimensional MFCC features.

We used the kNN classifier and Random Forest classifier under the default parameter configuration to make predictions on the above five data sets respectively. In order to obtain relatively accurate prediction results, all experimental tests were run on the same computer. Each data set was performed ten predictions, and the average of the ten predictions was taken as the final prediction result to reduce the random influence. The final prediction results are shown in Table 3.

It is worth noting that because the kNN classifier and the Random Forest classifier had achieved significant prediction effects in the entire research system that this research belongs to, this article selected them to make predictions on each data set from the recognition angle. Specifically, the kNN classifier adopts the idea of majority voting in principle, and it has achieved the best overall prediction performance in the entire research system, while the Random Forest classifier adopts the ideal of ensemble learning, and has also achieved better prediction performance. This article comprehensively analyzes the prediction results of the two, and evaluates the processing effects of the five signal filtering methods from the recognition angle. The specific descriptions of the prediction results achieved by the two classifiers on the five data sets are shown in Table 4 and Table 5.

It can be seen from the above table that, when the kNN classifier and the Random Forest classifier were used to make predictions on the five data sets, respectively, the average prediction accuracy achieved by the two classifiers on the data set established from the sound signal filtered by Improved Spectral Subtraction are both higher than that achieved by Basic Spectral Subtraction, which are about 0.6% and 1.3% higher, respectively. The average prediction accuracy achieved by the two classifiers on the data set established from the sound signal that filtered by the thirty-atom Sparse Decomposition is equivalent to the fifty-atom Sparse Decomposition. The two classifiers both achieved the highest average prediction accuracy on the data set established from the sound signal that was filtered by Wiener filtering, being respectively 88.83% and 88.69%, which are significantly higher than that achieved by other four signal filtering methods. Therefore, from the recognition angle, Improved Spectral Subtraction based on multi-taper spectrum estimation is better than Basic Spectral Subtraction, which is consistent with the conclusion drawn from the signal angle. In addition, Wiener filtering has achieved the best filtering effect, and the obtained prediction accuracy was about 10% higher than other four signal filtering methods.

### 3.4. Comprehensive Analysis

**Figure 6:**
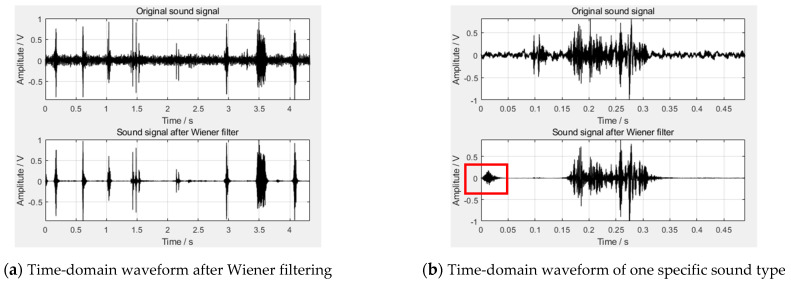
One sound signal filtered by Wiener filtering.

On the basis of analyzing and drawing preliminary conclusions from the signal angle and the recognition angle, respectively, we have to conduct a comprehensive analysis. From the signal angle, Improved Spectral Subtraction based on multi-taper spectral estimation has achieved the best signal filtering effect, while from the recognition angle, Wiener filtering has achieved the best signal filtering effect. Considering that Wiener filtering has achieved obvious performance advantages from the recognition angle, but its performance is very general from the signal angle, we have to consider it. We take one sound signal processed by Wiener filtering to draw a time-domain signal diagram, and intercept one specific sound type to draw the time-domain signal diagram, as shown in Figure 6.

It can be seen from Figure 6b that in the sound signal filtered by Wiener filtering there still exists a period of noise at the beginning, as shown by the red box in the figure. Therefore, when we calculate the SNR and RMSE of the sound signal processed by Wiener filtering, the calculation result was incorrect. This is also the reason why the processing effect of Wiener filtering is general from the signal angle. It should be noted that there are two main reasons for this noise. The first aspect is that the estimated value given by Wiener filtering spectrum estimator at the initial time is deviated from the true value, so a period of error noise will be generated. The second aspect is that the working principle of the Wiener filtering spectrum estimator is constantly adjusting the initial estimated value of the signal to approximate the true value. However, at the initial time, it is in a state of adjustment, which is unbalanced, and this noise is also a manifestation of the initial unstable state. In the signal processing field, this noise is usually not taken into consideration. Therefore, we filtered out the leading noise in the sound signal after Wiener filtering and recalculated the SNR and RMSE of the intercepted ten sound signals. Finally, the new average SNR was 5.6108, and the new RMSE was 0.0551. It can be seen that the newly calculated SNR was higher than Improved Spectral Subtraction, and the newly calculated RMSE was close to Improved Spectral Subtraction and was significantly better than the other four signal filtering methods. Considering that the larger the SNR or the smaller the RMSE of the sound signal before and after signal filtering, the better the filtering effect, at this time we can conclude that the filtering effect achieved by Wiener filtering and Improved Spectral Subtraction was equivalent from the signal angle. Therefore, when we comprehensively considered from both the signal angle and the recognition angle, the processing effect of Wiener filtering is the best. Moreover, the entire research system described in this study is ultimately aimed towards building a classifier with good prediction performance to predict specific sound types and their numbers in a fixed-duration sound signal, while Wiener filtering has an absolute performance advantage when comparing from the recognition angle. Finally, this research determined that Wiener filtering was the best signal filtering method in this article.

## 4. Discussion

This research has made a comprehensive consideration from both the signal angle and the recognition angle, and found that Wiener filtering is the best method for broiler sound signal filtering. In fact, in the signal processing field, adaptive filtering methods are often used to filter noisy signals. This research also used this to perform exploratory research in an early stage, but the actual filtering effect is not good. Specifically, when evaluating it from both the signal angle and the recognition angle, it performed even worse than Basic Spectral Subtraction (the worst performer in this article). Sparse Decomposition selected in this research is specifically an Orthogonal Matching Pursuit based on Genetic Algorithm, which is developed on the basis of the MP algorithm and OMP algorithm. The signal Sparse Decomposition is essentially to express the signal in a concise and clear mathematical language, that is, it is hoped that the number of atoms used in the signal is as low as possible, and the signal is not distorted. As such, Sparse Decomposition (or Sparse Representation) is essentially an optimization problem, and the greedy algorithm is a common method of solving this type of problem. The MP algorithm is the earliest and representative greedy algorithm [38,39]. Its main idea is to use as few atoms as possible to linearly represent the input signal from a given over-complete dictionary based on a certain similarity measurement criterion, so as to achieve an approximation of the input signal. The disadvantage of the MP algorithm is that in the optimization process, an atom may be repeatedly selected, which leads to poor convergence of the algorithm [40]. It should be noted that the direction selection of the MP algorithm is sub-optimal, not optimal. This is because the residual is only perpendicular to the current projection direction, so in the next projection, it is likely to be projected to the original direction again [41]. In order to solve the problem of selecting the optimal projection direction of the MP algorithm, the author further introduced the Orthogonal Matching Pursuit (OMP) algorithm.

The OMP algorithm was proposed by Y.C. Pati and R. Rezaiifar [42]. Compared with the MP algorithm, the advantage of the OMP algorithm is that it first processes all the atoms through the Gram-Schmidt regularization method, and then uses the residual signal to project the selected atoms, which ensures that the result of each iteration is the global optimal solution. However, from the analysis of the decomposition time, because the OMP algorithm needs to be orthogonalized every time before projecting on the selected atom, the calculation amount is relatively large. In this research, when the OMP algorithm was selected to carry out the preliminary research, although the signal filtering effect was improved, the calculation efficiency was still very low. Thus, this research introduced a Genetic Algorithm to optimize the OMP algorithm and formed a GA-OMP algorithm for signal sparse decomposition. The Genetic Algorithm proposed by John Holland is a bionic algorithm whose core idea is to simulate the process of mutation and natural selection. Through natural selection, it weeds out bad solutions. Through the reproduction mechanism, the optimal solution is retained. A Genetic Algorithm is an efficient parallel global search algorithm which defines a parameter to be optimized, the absolute value of the signal or signal residual, and the inner product of the atom as the fitness function. This is a good solution to the problem of finding the optimal matching atom in each iteration of the OMP algorithm when the signal is sparsely represented.

In addition, in the signal filtering method of this article, we choose the first method to determine the iterative stop condition of Sparse Decomposition. The author checked the relevant literature and found that Cheng et al. [14] had also conducted the research on broiler signal filtering. In her research, when Sparse Decomposition is used for signal filtering, the second method is selected to determine the iterative stop condition, and the achieved signal filtering effect is excellent. In the next step, the author considers it, and compares it with the existing Wiener filtering and the sub-optimal Improved Spectral Subtraction based on multi-taper spectral estimation to analyze whether there is a greater performance improvement.

Moreover, when analyzing the five signal filtering methods from the signal angle, the calculation results are as shown in Table 1. Among them, part of the SNR calculated by Sparse Decomposition has a negative value. For example, the SNR calculated from the NO.1 filtered sound signal that uses the thirty-atom Sparse Decomposition is −0.7658. This shows that the quality of the sound signal filtered by the thirty-atom Sparse Decomposition is even worse than that without signal filtering. This indicates that Sparse Decomposition method that uses the current atoms not only sparse the noise in the noisy signal, but also sparse the noise-free signal. That is to say, the noise in this sound signal was relatively small, so in the sparse process, only a small part of the noise was processed, and most of it was the processing of the noise-free signal. When adjusting the number of atoms, this situation will be effectively suppressed. For example, when using fifty-atom Sparse Decomposition to filter the NO.1 filtered sound signal, the calculated SNR is adjusted to −0.1859, which is significantly improved compared to the previous value. The same situation also appeared in the NO.2 and NO.2 filtered sound signals. In fact, the author tried to increase the number of atoms to eighty during the research process. Although the SNR obtained was no longer negative, the degree of reduction of the noisy signal was already high, and the signal filtering effect was basically insignificant. Thus, the author will conduct an in-depth study on this part later.

After obtaining the best method for broiler sound signal filtering, for the next step the author will use this as a basis to carry out research on broiler health monitoring with reference to the classification algorithm in machine learning to improve the broiler welfare. Specifically, the author will extract multiple classification features that can distinguish between crows, coughs, purrs, and flapping wings from the filtered broiler sound signals, and build a machine learning data set. Classifiers based on different classification algorithms will be trained on the data set, the inherent parameters of the best performed classifier will be optimized, and the final classifier for the classification of specific sound types (crows, coughs, purrs, and flapping wings) will be obtained. In this way, for a piece of broiler sound signal recorded in the broiler farm, after signal filtering and feature extraction, a data set to be predicted can be formed. We can apply the classifier to make the predictions, and then obtain the specific number of crows, coughs, purrs, and flapping wings in each signal. By comparing the ratio of the number of coughs to the number of all sound types, the cough rate used to judge broiler health is obtained, and the judgment of broiler health is completed. This is in principle consistent with the fact that the breeders judge the cough frequency of the broilers in the current area on the spot. According to the judgment result, protective measures can be taken to improve broiler welfare.

## 5. Conclusions

Research on evaluating the filtering method for broiler sound signals from multiple perspectives in this article was conducted from both the signal angle and the recognition angle, and the filtering effects of Basic Spectral Subtraction, Improved Spectral Subtraction based on multi-taper spectrum estimation, Wiener filtering, thirty-atom Sparse Decomposition, and fifty-atom Sparse Decomposition were evaluated. From the signal angle, it can be concluded that Improved Spectral Subtraction based on multi-taper spectrum estimation has obtained the highest average SNR of 5.5145 and the smallest average RMSE of 0.0508. From the recognition angle, it can be concluded that the kNN classifier and Random Forest classifier have achieved the highest average prediction accuracy on the data set after Wiener filtering, which were 88.83% and 88.69%, respectively. Taking into account the unavoidable presence of leading noise in the sound signal processed by Wiener filtering, and after removing the leading noise, Wiener filtering obtained the highest average SNR of 5.6108, obtained a new RMSE of 0.0551, and performed well in the five signal filtering methods. Therefore, a comprehensive analysis was finally carried out from both the signal angle and the recognition angle, and Wiener filtering was determined as the best method for broiler sound signal filtering. This research is the basis for the author to carry out follow-up systematic research and has important practical value for realizing the monitoring of broiler health in the farm. At the same time, the research results can be extended and applied to the research on the detection and processing of livestock and poultry sound signals.

## Figures and Tables

**Table 1:** SNR and RMSE of five signal filtering methods.

Signal Number	BSS		ISS		WF		SD/Thirty Atoms		SD/Fifty Atoms	
	SNR	RMSE	SNR	RMSE	SNR	RMSE	SNR	RMSE	SNR	RMSE
1	1.7691	0.1201	3.5719	0.0976	1.4732	0.1243	−0.7658	0.1513	−0.1859	0.0415
2	3.8273	0.0435	5.8593	0.0344	1.8472	0.0546	−1.6833	0.0768	−0.1243	0.0639
3	2.1030	0.0534	3.7349	0.0442	0.9655	0.0608	3.1437	0.0482	3.3780	0.0469
4	2.6685	0.1023	3.8642	0.0901	2.4198	0.1064	2.3336	0.1168	2.8879	0.1095
5	1.7754	0.0570	3.5703	0.0464	0.9126	0.0629	2.6798	0.0522	4.1521	0.0441
6	2.5100	0.0333	3.7005	0.0291	1.6632	0.0367	1.1763	0.0374	3.6884	0.0280
7	5.0925	0.0312	9.1441	0.0196	4.0548	0.0359	−1.2686	0.0529	−0.2485	0.0466
8	3.4524	0.0939	5.4609	0.0745	3.0098	0.0988	1.9618	0.0210	2.8147	0.1096
9	6.1313	0.0308	8.6555	0.0230	4.8939	0.0355	0.2187	0.0586	0.5416	0.0564
10	4.3272	0.0720	7.5838	0.0495	5.9567	0.0597	1.1845	0.1085	1.7164	0.1021
Mean value	3.3657	0.0638	5.5145	0.0508	2.7197	0.0676	0.8981	0.0724	1.8620	0.0649

**Table 2:** Specific description of five data sets corresponding to five signal filtering methods.

	Label	Crow	Cough	Purr	Flapping Wing	Total Numbers
Method						
BSS		3182	683	6513	3736	14,014
ISS		2308	717	6863	3147	13,125
WF		3004	735	5194	3813	12,746
SD/Thirty atoms		2666	1160	10,715	2435	16,976
SD/Fifty atoms		2466	799	9567	2424	15,256

**Table 3:** Ten prediction accuracies achieved on the five data sets.

Test Times	BSS		ISS		WF		SD/Thirty Atoms		SD/Fifty Atoms	
	kNN	RF	kNN	RF	kNN	RF	kNN	RF	kNN	RF
1	78.77	77.81	77.81	80.57	89.66	88.25	69.68	75.77	67.99	75.73
2	77.40	78.93	79.74	79.96	89.01	89.37	70.04	74.75	69.96	75.70
3	78.09	78.17	78.62	78.06	89.45	88.22	68.68	75.87	69.96	76.45
4	76.74	78.62	77.88	79.25	88.51	88.14	68.35	75.97	70.02	76.14
5	77.93	77.98	79.52	79.99	89.19	87.99	69.35	76.22	68.84	75.70
6	77.55	77.10	76.84	79.86	88.51	89.43	68.66	75.79	69.85	75.75
7	78.54	77.53	77.31	79.89	88.54	88.62	70.02	76.18	69.39	75.16
8	78.69	77.93	79.17	78.16	88.51	89.35	69.49	75.63	69.83	75.75
9	77.20	78.31	78.93	79.10	87.88	88.90	70.02	76.61	69.89	76.69
10	78.26	77.93	79.02	78.77	89.01	88.59	69.74	77.03	69.78	75.20
Mean value	77.92	78.03	78.48	79.36	88.83	88.69	69.40	75.98	69.55	75.83

**Table 4:** Specific descriptions of the prediction results achieved by the kNN classifier on the five data sets.

	Predictions	Total Number of Data	Number of Data Predicted Correct	Number of Data Predicted Incorrect	Predict Accuracy/%
Method					
BSS		14,014	10,920	3094	77.92
ISS		13,125	10,301	2824	78.48
WF		12,746	11,322	1424	88.83
SD/Thirty atoms		16,976	11,781	5195	69.40
SD/Fifty atoms		15,256	10,611	4645	69.55

**Table 5:** Specific descriptions of the prediction results achieved by the Random Forest classifier on the five data sets.

	Predictions	Total Number of Data	Number of Data Predicted Correct	Number of Data Predicted Incorrect	Predict Accuracy/%
Method					
BSS		14,014	10,935	3079	78.03
ISS		13,125	10,416	2709	79.36
WF		12,746	11,304	1442	88.69
SD/Thirty atoms		16,976	12,898	4078	75.98
SD/Fifty atoms		15,256	11,569	3687	75.83

## Data Availability

Some or all data, models, or code that support the findings of this study are available from the corresponding author upon reasonable request.
